# Randomised social-skills training and parental training plus standard treatment versus standard treatment of children with attention deficit hyperactivity disorder - The SOSTRA trial protocol

**DOI:** 10.1186/1745-6215-12-18

**Published:** 2011-01-21

**Authors:** Ole Jakob Storebø, Jesper Pedersen, Maria Skoog, Per Hove Thomsen, Per Winkel, Christian Gluud, Erik Simonsen

**Affiliations:** 1 Child Psychiatric Daytime Clinic, Child and Adolescent Psychiatric Centre, Region Zealand, Holbaek, Denmark; 2 Psychiatric Research Unit, Region Zealand, Roskilde, Denmark; 3 Copenhagen Trial Unit, Centre for Clinical Intervention Research, Department 3344, Rigshospitalet, Copenhagen University Hospital, Copenhagen, Denmark; 4 Psychiatric Hospital for Children and Adolescents, Risskov, Denmark; 5 Cochrane Hepato-Biliary Group, Copenhagen Trial Unit, Centre for Clinical Intervention Research, Department 3344, Rigshospitalet, Copenhagen University Hospital, Copenhagen, Denmark; 6 Faculty of Health Sciences, University of Copenhagen, Copenhagen, Denmark; 7 Institute of Psychology and Education, Roskilde University, Denmark; 8 Aarhus University, Clinical Institute, Child and Adolescent Hospital, Centre for Research and Development, 8000 Aarhus, Denmark

## Abstract

**Background:**

Children with attention deficit hyperactivity disorder (ADHD) are hyperactive and impulsive, cannot maintain attention, and have difficulties with social interactions. Medical treatment may alleviate symptoms of ADHD, but seldom solves difficulties with social interactions. Social-skills training may benefit ADHD children in their social interactions. We want to examine the effects of social-skills training on difficulties related to the children's ADHD symptoms and social interactions.

**Methods/Design:**

The design is randomised two-armed, parallel group, assessor-blinded trial. Children aged 8-12 years with a diagnosis of ADHD are randomised to social-skills training and parental training plus standard treatment versus standard treatment alone. A sample size calculation estimated that at least 52 children must be included to show a 4-point difference in the primary outcome on the Conners 3^rd ^Edition subscale for 'hyperactivity-impulsivity' between the intervention group and the control group. The outcomes will be assessed 3 and 6 months after randomisation. The primary outcome measure is ADHD symptoms. The secondary outcome is social skills. Tertiary outcomes include the relationship between social skills and symptoms of ADHD, the ability to form attachment, and parents' ADHD symptoms.

**Discussion:**

We hope that the results from this trial will show that the social-skills training together with medication may have a greater general effect on ADHD symptoms and social and emotional competencies than medication alone.

**Trial registration:**

ClinicalTrials (NCT): NCT00937469

## Background

Attention deficit hyperactivity disorder (ADHD) affects 3% to 5% of all children [1]. The main ADHD symptoms consist of problems with attention, impulsiveness, and hyperactivity [2,3]. Pharmacological treatment of children and adolescents with ADHD has beneficial effects on these symptoms in about 80% of the patients [4-12]. However, many children and adolescents with ADHD also frequently have difficulties regarding language, learning, anxiety, and interaction with parents and teachers. These difficulties can be severe, and there is little evidence that medication has an effect on these outcome measures [13-17]. Children with ADHD also have an increased risk of developing personality disturbance and possibly psychotic conditions, abuse of drugs or alcohol, and criminality [18-24]. Comorbid disorders in children with ADHD often include behavioural disorders, depression, anxiety, tics, motor skill development disturbance, learning difficulties, and verbal and cognitive difficulties [25,26].

### Social-skills training

Social-skills training aims to develop, improve, and maintain the individual's social skills. This is achieved by teaching how to regulate verbal and nonverbal behaviours involved in social interactions and in compliance with social norms [27,28]. The main elements in social-skills training include training of social skills and efforts to change the individual's cognitive assessment of the 'social world' and to develop these cognitive skills (Fohlmann AH: E-mail correspondence in April 2009). Concretely, the training focuses on teaching the children to read the subtle cues in social interactions, such as learning to wait for their turn or knowing when to shift topics during a conversation and being able to recognise the emotional expressions of others. A few randomised clinical trials suggest that social-skills training may help children with ADHD [29-31]. Other studies indicate that only some children benefit from social-skills training, possibly due to lack of parental engagement in the training [32]. Like with medical treatment, the effects of social-skills training do not always appear to endure over time. It is even argued that social-skills training groups can have a negative effect on children with behavioural problems because the aggressive and restless behaviour in itself can limit the ability to learn social skills [33]. We have been unable to identify any meta-analyses or systematic reviews on the topic.

### Abilities in forming attachments

A child's ability to form attachments is developed in early childhood through interaction with primary caregivers. Different forms of attachment are secure, insecure dismissing, insecure preoccupied, and disorganised. It is assumed that these different forms of attachment will influence the outcome of social-skills training. A connection between early disorganised attachment and later ADHD has been demonstrated by Punto et al. [34], who followed children from birth to 7 years of age.

The primary aim of the SOSTRA trial is to examine the effect of the combination of social-skills training and parental training plus standard treatment versus standard treatment alone in children with ADHD and their families on the outcome measures of ADHD core symptoms, social skills, and the attachment between the child and the parents.

## Methods/Design

**Figure 1:**
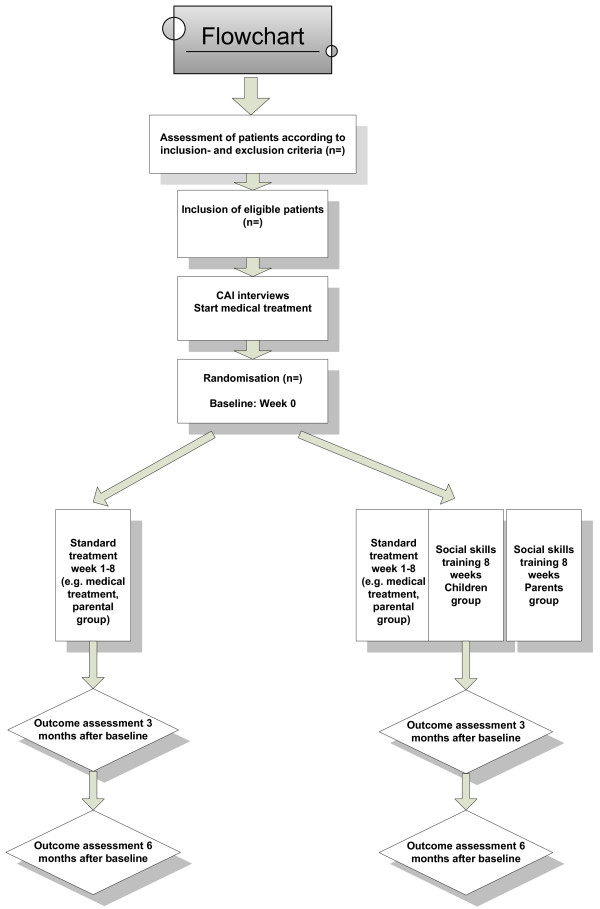
**A flow chart of the SOSTRA design**.

Children aged 8-12 years with a diagnosis of ADHD and their parents are randomised to the combination of social-skills training and parental training plus standard treatment versus standard treatment alone. The trial flowchart is shown in Figure 1. The trial is a randomised two-armed, parallel group, assessor-blinded trial. The children will be examined at entry, 3 months, and 6 months after randomisation.

### Experimental intervention

Children and parents randomised to the experimental intervention are included in 1 of 4 identical 8-week social-skills treatment programs with 12 to 16 participants per program. Here, the children are offered weekly social-skills training sessions of 90 minutes in duration. During that time, the parents attend parental training. Each group has two therapists who have been trained in management of social-skills training at Langager School in Aarhus [35]. The experimental interventions are thoroughly described in a manual, and each session with the children will be video recorded. The group sessions are planned to be on the same day and at the same time every week, just as the structure and the agenda of the sessions will be the same. The experimental intervention program is organized similarly to other randomised trials [29-31] and with supervision from the Langager School. Different methods of teaching the children social skills are used, all of which have proved successful in other social-skills programs [36]. These include didactic instructions, work with symbols (e.g. dolls), role-play, creative techniques, physical exercises, music, story reading, games, and movies. Each session has a theme, such as self worth, nonverbal communication, feelings, impulse control, aggression management, conflict resolution, and problem solving. The themes are connected to the trials outcome measures of ADHD core symptoms, social skills, and the attachment between the child and the parents.

Social skills are based on the broad area of cognition and emotions. The iceberg model (Division TEACCH, North Carolina, USA) emphasizes the importance of considering each child's problems and resources distinctly and incorporating these in the training programme. For instance, some children may need more sessions with visualization techniques to better learn the management of aggression. When they get better at managing their aggression, they will also be able to improve their social skills. The social-skills experimental intervention will focus on strengthening the ability of the children to control themselves and start a self-help process. For the parents, efforts will be directed at helping them develop strategies to assist their children in controlling their impulses. It is important that this training equips the children and parents with the skill to cope better and to reverse the bad circles. The efficacy of the experimental intervention will be assessed by improvements in ADHD symptoms and social skills per se or by assessing psychological functioning on a broader aspect, including the quality of peer relationships and emotional competencies.

Efforts must be made to create a safe environment in the group. The children should feel safe enough to play and experiment with exploring their own and other people's understanding of them along with understanding the other participants and the different topics in focus. The assignments must be clear and simple. The educational style in the groups will take into account the children's special cognitive difficulties; accordingly, the structure in each session will be predictable. This is secured by regular items on the agenda, which are written on the blackboard every time: Opening round--what has happened since the last time? — revision of the previous session; homework from previous session; presentation/education; role play/creative activities; new homework; closing round.

The therapists must be clear and direct but not confrontational or critical. Weight is attached to empathy, positive reinforcement, and a curious 'non-knowing' mentalizing attitude. A relaxed atmosphere with room for humour is the aim.

The therapists who are responsible for the children's group in the experimental intervention arm are also responsible for the parents' group in the standard treatment arm. This risk of bias will be limited by writing the detailed manual that will describe the content in each group. Content forms are to be completed by the therapists after each group sessions and will function as a control of the content of the sessions given.

In the parental groups, the themes from the children's groups will be presented and discussed. Likewise, the children's homework will be discussed and parents are encouraged to discuss their specific problems with their children and there will be an exchange of experiences among the participants.

### Standard treatment

**Figure 2:**
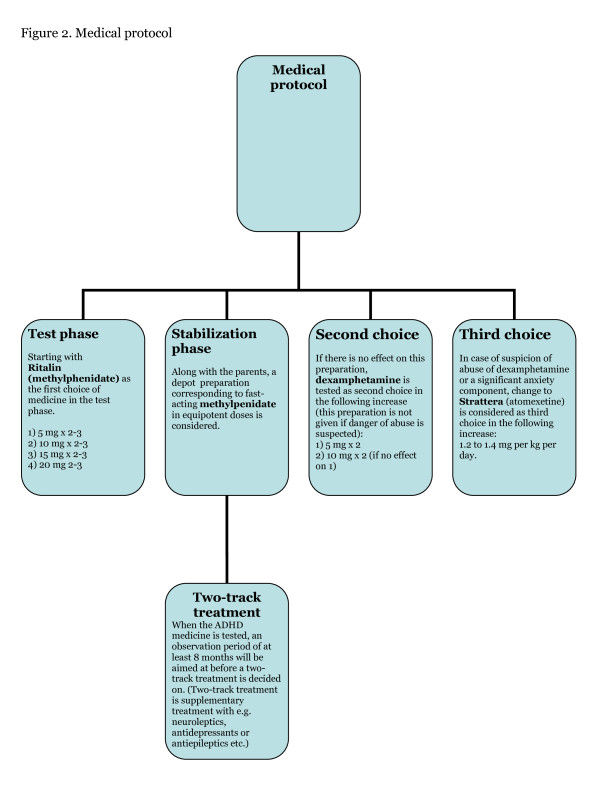
**Medical protocol**.

The standard treatment offered to both the experimental group and the control group encompasses the normal practice regarding ADHD patients at the Child Psychiatric Daytime Clinic in Holbaek. The overall objective is to secure compliance with the treatment, which means that the team attaches importance to building an alliance with the family, creating safety, and ensuring that the family receives sufficient counselling, psychological education, and support to enable them to be more confident and autonomous regarding the problems and challenges of having a child with an ADHD. After assessment and confirmation of the ADHD diagnosis, the family is offered medical treatment for the child. The medical protocol is shown in Figure 2. Medical treatment is always preceded by a physical examination; the somatic condition of the child is examined and an individually adapted neurological examination is performed. The physician informs the parents of the advantages and disadvantages of medical treatment. The family is asked to contact the clinic within a week of medical treatment initiation to report on the child's progress. From this meeting, it is decided whether the dosage regimen is satisfactory. All children are examined again after 1 month of treatment; positive and adverse effects are evaluated and educational counselling is given. If the child has gained weight, nutrition advice is given; if the child has developed sleeping problems, a special duvet can be borrowed or medication can be prescribed. The standard treatment involves a parent group where the parents meet three times during the 8-week trial period. In addition to general information about ADHD, focus is placed on different aspects related to the disorder, such as the child's relationship with siblings and peers. Talks will be given by visiting adults who have been diagnosed with ADHD. They talk to the group about their experiences of living with ADHD, having children, and making everyday life function.

### Screening and recruitment of participants

The children are those referred to the Child Psychiatric Clinics in Holbaek and Roskilde with an ADHD diagnosis. They are screened according to the inclusion and exclusion criteria (Table 1). The parents will sign a written informed consent in the first meeting in the clinic. The Schedule for Affective Disorders and Schizophrenia for School-aged Children (K-SADS) will be used in the baseline assessment. This semi-structured interview includes algorithms from the Diagnostic and Statistical Manual of Mental Disorders (DSM-IV) in children and adolescents [37]. The child will also be screened for autism with the two Social Communication Questionnaires (SCQ) completed by the parents. The child will be excluded from the trial if there is a cut-off score above 15 on both the SCQ questionnaires [38]. The parents also complete the Adult Self-Report Scale (ASRS), which is a screening for adult ADHD symptoms [39]. The child's teachers will be asked to complete the Strength and Difficulties Questionnaire (SDQ) [40]. The child will be tested with the Children Attachment Interview (CAI) [41] before any medical treatment is initiated. The children who have not been assessed by the Wechsler Intelligence Scale for Children (Wisc-3 test) during the last 3 years will be tested with the Wisc-3 test by psychologists from the Clinic [42].

**Table 1:** Criteria for inclusion and exclusion in the SOSTRA trial

**Inclusion**:	**Exclusion**:
**1) **The parents should be interested in taking part in parental groups in Child Psychiatric Clinic in Holbaek.	Patients with the following diagnoses according to DSM IV: **1)** Schizophrenia: 295.30 (Paranoid type); 295.10 (Disorganized type); 295.20 (Catatonic type); 295.90 (Undifferentiated type); 295.60 (Residual type); 295.70 (Schizoaffective Disorder); 297.1 (Delusional Disorder); 298.8 (Brief Psychotic Disorder); 297.3 (Shared Psychotic Disorder); 298.9 (Psychotic Disorder Not Otherwise Specified).

**2) **The patients (and the parents) must understand and speak Danish language to an extent where a translator is not needed in order to be able to complete the assessment and the treatment.	**2) **Children with autism according to DSM IV: 299.00 (Autistic Disorder); 299.10 (Childhood Disintegrative Disorder); 299.80 (Asperger's Disorder), *or a cut of score on both the SCQ questionnaires above 15.*

**3) **The patients' parents must give informed consent to participate in the trial.	**3) **Violent and criminal youngsters.

**4) **The child must be between 8 to 12 years old by the time of the start of the assessment.	**4**) Children with a total verbal and nonverbal intelligence quotient below 80 according to WISC III

**5) **Both boys and girls can participate.	**5) **Strong resistance from the child against participating.

**6) **Children with a total verbal or nonverbal intelligence quotient over 80 according to the WISC III.	**6) **Previous started medical treatment for ADHD.

**7) **The children must fulfil research criteria for the diagnosis ADHD according to DSM IV (1994): 314.00, 314.01, 314.02. or 314.9.	**7**) Lacking informed consent.

**8) **The parents must consent in medical treatment for their child and there must be clinical indication for medical treatment.	

We estimate that 20 children will be assessed per treatment program (four identical programs are scheduled). This is the number that we believe is needed to produce approximately 12 to 16 participants for randomisation.

### Outcome measures

All outcomes will be assessed before the interventions starts and at 3 months and 6 months after randomisation. The primary outcome measure is an assessment of ADHD core symptoms. The secondary outcome measures are an assessment of the children's social skills. The tertiary outcomes are an assessment of the attachment between the child and the parents and an assessment of the parents own ADHD symptoms (Table 2).

**Table 2:** Outcome measures in the SOSTRA trial

*Primary outcome:*	*Measured by:*
ADHD symptoms.	Conners' 3^rd ^Edition subscale 'hyperactivity-impulsivity' (teacher rated).

***Secondary outcomes:***	
Social skills.	Conners' CBRS subscale 'social problems' (teacher rated) and by Conners' 3^rd ^Edition subscale: 'peer relations' (teacher rated).
Aggressive behaviour.	Conners' CBRS subscale: 'aggressive behavior' (teacher rated).
Emotional distress.	Conners' CBRS subscale: 'emotional distress' (teacher rated).
Executive functions.	Conners' 3^rd ^Edition subscale: 'executive functioning' (teacher rated).
Academic performance.	Conners' CBRS: subscale: 'academic performances' (teacher rated).

***Tertiary outcomes:***	
Social skills and symptoms of ADHD measured in relation to attachment.	Children Attachment Interview.
Improvements in the ability to form attachments.	Children Attachment Interview.
Social skills and symptoms of ADHD measured in relation to parental ADHD symptoms.	Adult Self Report Scale Symptom Checklist.

### Assessment instruments

• **CAI **[41]. The CAI focuses on the child's experiences of his/her own present relevant relations and measures the child's view of his/her attachment figures' accessibility and sensitivity through the exploration of the inner object representations. The test consists of 19 questions that ask the child to recall experiences with his/her important attachment figures, especially at times where the child has been sad, anxious, or ill. The interview will be video recorded, and a scoring weight will be attached both to verbal and nonverbal statements. The test can assign the children to 1 of 6 possible attachment categories. In this study, the children are given 1 of the following four attachment categories: *secure attachmen*t, *insecure*, *disorganized/secure, or disorganized/insecure attachment*. This test can be used for children aged 6 to 14 years. (Danish version).

• **K-SADS clinical diagnostic interview **[37]. This test has been translated into Danish by Dorthe Janne Petersen and Niels Bilenberg. It is an internationally known diagnostic interview system, referred as Schedule for Affective Disorders and Schizophrenia for School-aged Children, Present and Lifetime Version (K-SADS-PL). The interview is used to diagnose children aged 6 to 18 years. Using the interview makes it possible to classify child and youth psychiatric diagnoses according to the DSM-III-R and DSM-IV-systems.

• **Conners CBRS Teacher **[43]. This questionnaire has been translated into Danish by psychologist Ole Jakob Storebø and psychologist Kirsten Bach in collaboration with Dorte Damm, Per Hove Thomsen, and the Dansk Psykologisk Forlag. This is an internationally approved instrument, which is used on children aged 6 to 18 years. The instrument has strong psychometrically qualities and measures behaviour, school performance, and emotional and social abilities.

• **Conners 3**^**rd **^**Edition Teacher **[44]. This questionnaire has been translated into Danish by psychologist Ole Jakob Storebø and psychologist Kirsten Bach in collaboration with Dorte Damm, Per Hove Thomsen, and Dansk Psykologisk Forlag. This is an internationally approved instrument, which is used on children aged 6 to 18 years. The instrument has strong psychometrically qualities and measures ADHD core symptoms, behaviour, and emotional and social abilities.

• **ASRS Symptom Checklist **[39]. This questionnaire covers the 18 DSM-IV-TR criteria for ADHD. It is used on adults. According to the American background material, 6 of the 18 questions have been identified as the most predicative of symptoms in relation to ADHD. These 6 questions form the basis of the ASRS v1.1 screening instrument and represents section A of the symptom checklist. Section B of the symptom checklist contains the remaining 12 questions. (Danish version).

• **SCQ **[38]. This is a screening instrument for autism and autism-spectrum disturbance among children aged four years and older, and is filled out by the parents.

• **SDQ **[40]. This is a brief behavioural questionnaire for children aged 3 to 16 years, and is filled out by the teacher.

• **Wisc 3 **[42]. This is a test used to evaluate intelligence and cognitive functions among children aged 6-16 years.

### Randomisation

Central randomisation is performed by the Copenhagen Trial Unit (CTU) with computer generated, permuted randomisation sequence with unknown block size for the investigators. A research secretary will call the CTU and providing a personal pin code, patient number, and the stratification variables of sex (female/male) and comorbidity (yes/no). Then the randomisation will be announced.

### Blinding

The interventions are not blinded to participants, parents, treating physicians, or personnel in the clinic. However, the outcome assessor of the primary and secondary outcomes is the teacher, who is kept blinded of the child's allocated intervention. The involved parties (the parents and children) are instructed not to inform the teacher of the allocation. To secure integrity of trial data, the principal investigator will collect the questionnaires blind to the intervention. Blinded data will be handed over to the CTU, which will be in charge of data entry and statistical analyses blinded to intervention. Standardised procedures will be assured.

### Sample size

The sample size is calculated on the basis of a type I error (α) of 5% and a type II error (β) of 20%, thus a power of 80%, and an allocation ratio of 1:1. With a clinically relevant difference of a score of 4 between the experimental intervention group and the control group on the Conners 3^rd ^Edition Rating Scale 'hyperactivity-impulsivity' sub index (primary outcome) and an assumed standard deviation of 5 on the same scale [45,46], a sample size of 26 participants in each group is needed. This corresponds to a total of 52 participants to be randomised. In case of missing follow-up data (>5%), multiple imputations will be conducted (see below).

### Statistical analysis

#### Intention-to-treat and per-protocol analyses

The statistical analysis of the outcomes will be based on the 'intention-to-treat' principle, i.e. all randomised participants will be included in the analysis in the intervention group to which they were randomized, irrespective of how much of the intervention they have received. Per-protocol analyses will be conducted secondarily for the participants who have completed 50% or more of their randomised intervention.

According to the ICH Harmonised Tripartite Guideline, Statistical Principles for Clinical Trials E9 Analysis of Drug Trials [47], the analyses will primarily be conducted with adjustment for stratification variables and will secondarily be conducted without adjustment for stratification variables.

#### Statistical analysis plan

The analysis of each outcome measure investigates if the outcome measure changes (increases or decreases) significantly over time in one intervention group as compared with the other one. The group coding is concealed for the statistician. Provided the outcome measure is a primary or secondary one, it will be investigated to determine if the effect depends on each of the tertiary outcome measures as measured at entry. If this is the case, *post hoc *explorative subgroup analyses prompted by the result may be conducted.

To deal with the multiplicity problem, the hypotheses will be ordered into families of hypotheses, and these families will in turn be ordered into a hierarchy of families. The general multistage gate keeping procedure of Dmitrienko et al. (2008) will then be applied [48]. The two-sided significance level will be 0.05. The gate keeping will be parallel, and the hypotheses will be organised into the following families:

1. Hypotheses related to the effect on the primary outcome measure.

2. Hypotheses related to the effect on the secondary outcome measures.

3. Hypotheses related to the effect on the tertiary outcome measures.

Once none of the null hypotheses in a family are rejected, the procedure stops and the rest of the null hypotheses are accepted. However, the raw P-values of the remaining tests will be calculated and presented as the results of *post hoc *analyses for hypothesis generating purposes.

The complete analysis of each outcome measure is the same for the primary and secondary outcome measures. The outcome measures is set to be M (continuous variable), the indicator of intervention to be I (binary categorical variable), time to be t (a continuous variable (0, 3, or 6 months)), and the tertiary outcome at baseline to be O3-baseline (it will be dealt with as a nominal variable in this context). The possibility that the value of M increases between month 3 and 6 is a real one. Therefore, the full model may not necessarily be a linear model in t. Consequently, we will include the quadratic component t2 in the full model. However, the final choice of model may be tempered by the impression obtained from the inspection of the marginal mean values.

The mixed model repeated measures method will be used. The model statement without the O3-baseline included will be specified as follows:

$$\text{M}=\text{intercept}+\text{aI}+\text{bt}+{\text{ct}}^{2}+\text{dIt}+{\text{eIt}}^{2}$$

where a thru e are coefficients in the model. This model will test if the mean level as well as the linear and the quadratic effect of time differ significantly between the two interventions. A sequential hypothesis testing is used, which is appropriate for polynomial models. Initially, four types of covariance matrices will be tested: compound symmetric, AR(1), AR(1) with heterogeneous variances, and unstructured. Using the Akaike and the Schwartz Bayesian criteria, the best covariance structure will be chosen. In the analysis of the continuous tertiary outcome measures (measurements at entry and after 6 months), compound symmetric and unstructured covariance structures will be compared. When the O3-baseline is included, the full model will be augmented by the main effect of O3-baseline and interactions between O3-baseline and previously included components containing I, the intervention indicator.

Each analysis will be repeated twice for comparison (see sensitivity analyses). Missing observations imputed by the multiple imputations (MI) method will be included the first time, and only 'complete cases' will be included the second time. However, the main analysis will be a mixed model analysis including all original values (without any imputed ones included).

The second tertiary outcome measure is an ordinal variable with four possible categories. The proportional odds model with the same type of model statement as explained above will be used. If the assumption of this model is not fulfilled, various types of ordinal regression (SPSS version 17) will be attempted and, if this fails, a multinomial model will be used. If more than 5% of the values are missing, two analyses will be made, one only including 'complete cases' and the other including MI values.

#### Prevention of missing values

The teachers will be personally informed of the questionnaires by the therapist, who will assess the behaviour of the children in school. This therapist will inform the teacher of the importance that every question is answered in the questionnaires. The teachers will receive the questionnaire, along with a letter, at entry and at 3 and 6 months after treatment. In this letter, the necessity to answer all of the questions will be emphasized, and the teachers will be encouraged to call the principal investigator in case of any queries. The principal investigator and research secretary will play active roles in contacting the teachers of the children with ADHD and ensuring data collection in case the teachers fail to return the questionnaires. After receiving the questionnaires, the principal investigator and research secretary will assess all of the responses. If they find any questions unanswered, they will contact the teachers within 1 week of receiving the questionnaires, ensure that the missing data is completed, and find out why those questions were left unanswered. A short 1-day course will be arranged after the final follow-up and after receiving the final questionnaires for all of the teachers whose students participated in the trial. The teachers will be informed about this course once they receive the questionnaires at the baseline. All children and their parents will be contacted by the ADHD team for a long period of time after the end of this treatment, often as long as several years; therefore, all children and their parents will be bound to ambulatory settings and this study.

#### Types of missing values

Table 2 shows the outcome measures. From the forms (Connor 3rd Edition or Connor CBRS), the results of questions are combined algebraically to produce a number that is treated as the result of a continuous variable (the outcome measure). In the forms, the answers to the questions pertaining to the child's behaviour during the previous month are ordinal values (not true (0), sometimes true (1), often true (2), practically true all the time (3)). It is presumed that whether an outcome measure is reported or not reported due to at least one question remaining unanswered within a completed form does not depend on the unobserved value of the outcome measure. This type of missing outcome measure result is referred to as a type 1 missing value.

Outcome measure results may also be missing because the corresponding form was not completed due to drop out or other causes (type 2 missing value). In this case, it is not safe to presume that the pattern of missing values is not related to the unobserved data. The potential impact of type 2 missing values is explored using a worst-case analysis (see below).

#### Statistical analysis of missing values

One approach for dealing with missing values is to use a mixed model for repeated measures (MMRM) in the statistical analysis (see statistical analysis). This model prevents bias only if the missing at random (MAR) assumption is fulfilled (the pattern of missing values is related to the observed data only). The results of the study are those obtained by this method when it is applicable for the analysis of the data (the outcome result of the patients follow a normal distribution with reasonable approximation in each of the 6 groups formed by the possible treatment by time combinations).

For comparison, the method of MMRM is supplemented by that of MI using the model variables and additional variables significantly related to the variables with missing values and/or the absence of these variables. The method used is the fully conditional specification method of SPSS (version 17.0). This is an iterative Markov chain Monte Carlo method that can be used when the pattern of missing data is arbitrary (monotone or non-monotone). The default number of iterations is used initially and then increased if the Markov chain has not converged. Prior to the MI, the distributions of the continuous variables are inspected to see if serious deviations from the normal distribution that need transformations are present. Constraints are set to restrict the range of imputed values of continuous variables so that they are plausible. In total, 10 imputed data sets are produced.

#### Sensitivity analyses

Two sensitivity analyses will be performed.

1. Parameter estimates obtained by complete case analysis, MMRM, and MI followed by MMCM on the imputed data sets will be compared [49].

2. A worst-case analysis of the effect of type 2 missing values will be conducted as follows. The group coded Effect group will be designated the group with a significant and beneficial effect on the outcome measure (say it decreases over time) as compared to the group coded No Effect group. Missing values will now be imputed as follows: A). Value(s) missing in the Effect Group: A single value missing is imputed with an average of the 2 observed values. Two values missing are both imputed by the third observed value. Three values missing are all imputed by the grand mean of the No Effect group. B). Value(s) missing in the No Effect-group: A missing 6-month value is imputed by the smallest observed 6-month value in the data. A missing baseline value is imputed by the largest observed baseline value in the data. A missing 3-month value is imputed by the average of 0 and 6-month values whether observed or imputed as explained above. This should minimize the response to intervention based on imputed values in patients from the Effect group and give a maximal negative linear response based on imputed values in patients from the No Effect group given the constraint that values more extreme than those observed must never be used.

MI will only be used if the missing values exceed 5%.

#### Group comparison at entry

To establish if participant characteristics at trial entry are relatively similar in the 2 intervention groups (thereby a low risk of selection bias and confounding), demographic data (sex and age) and other factors that can be expected to influence the primary outcomes will be presented in a table of the entry characteristics.

### Ethical considerations and regulatory approval

Participants will be informed of the trial in writing and orally; written informed consent will be obtained from the participant's principal caregivers. There are no apparent ethical problems since all participants are offered standard medical treatment, and standard control treatment in this population, further, there are no known disadvantages of social-skills training. Nevertheless, any adverse events of the intervention will be reported. The trial has obtained approval by the Regional Ethics Committee of Zealand (SJ-85) and is registered with the Danish Data Protection Agency (J. nr.2008-41-2613) and at ClinicalTrials.gov (NCT00937469).

## Discussion

This trial compares the effects of social-skills training groups supported by parental training plus standard treatment versus standard treatment alone on the outcome of core ADHD symptoms of hyperactivity and impulsivity. A secondary objective is to examine differences in the effect of the treatment in relation to the children's different abilities in forming attachments: secure, insecure, disorganized/secure, or disorganized/insecure attachment. The last objective is to examine differences in the effect of the treatment in relation to the degree of parents' symptoms of ADHD.

The results from this trial can greatly benefit children with ADHD because social-skills training may have a greater general effect on social and emotional competencies than medication alone. Additionally, the connection between the ability to form different attachments and the effect of the social-skills training can influence both the understanding and the treatment of the disease. One of the trial's strengths is that it is related to very important matters in the development of comprehensive treatment programs for ADHD children. Many children with ADHD have serious problems with peers because of their lack of emotional and social abilities. ADHD children are often lonely, and their social problems often lead to a vicious circle, which is difficult to break [16,17].

Other strengths of this trial are the measurement of attachment styles in children with ADHD and the comparison between the effect of the social-skills training and the attachment competencies. We do not know of other studies in which children with an ADHD diagnosis are tested with the CAI test. Punto et al. stated that there is a significant connection between disorganised attachment in early childhood and ADHD symptoms in the school-age period [34]. This shows the necessity for more research on the topic. The possible connection between attachment problems and ADHD is an interesting topic in the ethological discussion. It must also be assumed that these children need a form of treatment that focuses on their inability to form relationships and their social problems. With the SOSTRA trial, we aim for improved the treatment of children with ADHD. There is a greater tendency towards pure medical treatment for children with ADHD, which is reprehensible because children need a more comprehensive treatment.

The experimental intervention in this trial is relatively short (8 weeks) and is therefore not very costly. This will allow other child psychiatric units to incorporate social-skills training for children with ADHD. A limitation of this trial is the relatively small sample size, which could make it difficult to draw firm conclusions about the research questions. However, if successful, it will be indicative of further directions for research on this topic.

## Abbreviations

**ADHD**: Attention Deficit Hyperactivity Disorder; **ASRS**: Adult Self-Report Scale; **CAI**: Children Attachment Interview; **DSM-IV**: Diagnostic and Statistical Manual of Mental Disorders; **ICD-10**: International Classification of Diseases; **IQ**: Intelligence quotient; **K-SADS-PL**: Schedule for Affective Disorders and Schizophrenia for School-Age Children-Present and Lifetime version; **MTA**: Multimodal Treatment Study of ADHD; **SCQ**: Social Communication Questionnaire; **SDQ**: Strength and Difficulties Questionnaire; **SOSTRA**: Social Skills Training and Attachment; **WISC-III**: Wechsler Intelligence Scale for Children; **Conners CBRS**: Conners Comprehensive Behaviour Rating Scales.

## Competing interests

Per Hove Thomsen has received a fee for lecturing on behalf of NOVARTIS and UCB as well as lecturing and consulting on behalf of Eli-Lilly. He also serves on the advisory board of Eli-Lilly.

All the other authors declare that they have no competing interests.

## Authors' contributions

All authors contributed to the design of the trial. OJS drafted the manuscript. All authors contributed to the further review of the manuscript. All authors read and approved the final manuscript.
